# A Bioactive Lipid Nanoparticle Integrating Arachidonic Acid Enables High-Efficiency mRNA Delivery and Potent CAR-Macrophage Engineering

**DOI:** 10.3390/ijms26189199

**Published:** 2025-09-20

**Authors:** Jia Fu, Yanan Zhang, Yifan Lv, Ruilin Li, Hongchen Gu, Jingxing Yang

**Affiliations:** 1 School of Biomedical Engineering, Med-X Research Institute, Shanghai Jiao Tong University, Shanghai 200030, China; fujia0626@sjtu.edu.cn (J.F.); zhangyn@sjtu.edu.cn (Y.Z.); lyf1113@sjtu.edu.cn (Y.L.); lrl20020505@sjtu.edu.cn (R.L.); 2 Hefei Institute of Innovative Early Cancer Screening Technologies, Shanghai Jiao Tong University, Hefei 230000, China

**Keywords:** arachidonic acid, lipid nanoparticles, macrophages engineering, transfection efficiency, gene delivery

## Abstract

Genetic engineering of macrophages, particularly for chimeric antigen receptor macrophage (CAR-M) therapy, holds great promise for immunotherapy, yet is constrained by the challenge of efficient gene delivery into primary macrophages, which are notoriously resistant to transfection. While conventional strategies focus on optimizing the physicochemical properties of lipid nanoparticles (LNP), they often fail to overcome the intrinsic biological barriers of these cells. Here, we introduced a “bioactive nanocarrier” paradigm, hypothesizing that incorporating a cellular modulator directly into LNP structure can synergistically overcome these barriers. We designed and synthesized a novel LNP by integrating the pro-inflammatory fatty acid, arachidonic acid (ARA), as a functional structural component (ARA-LNP). Systematic optimization of the ARA content and mRNA payload revealed a formulation that achieves high transfection efficiency (83.76%) in primary M2-polarized bone marrow-derived macrophages (BMDMs), a cell type that recapitulates pro-tumoral phenotype in the tumor microenvironment. Leveraging this advanced delivery platform, we successfully generated HER2-targeting CAR-M that demonstrated potent and specific phagocytic activity against HER2-expressing tumor cells in vitro. This work presents a powerful strategy where the nanocarrier itself transiently modulates the target cell state to enhance gene delivery, providing a new design principle for engineering macrophages and other hard-to-transfect immune cells for therapeutic applications.

## 1. Introduction

Macrophages are central orchestrators of tissue homeostasis and immunity, capable of adopting diverse functional phenotypes in response to microenvironmental cues [1,2,3]. In cancer, however, this remarkable plasticity is frequently exploited by tumors, resulting in the predominance of tumor-associated macrophages (TAMs) with an M2-like, pro-tumoral polarization. These TAMs actively suppress anti-tumor immunity, promote angiogenesis, and facilitate invasion and metastasis, thereby constituting a major barrier to effective cancer therapy [4,5,6]. Consequently, reprogramming these pro-tumoral TAMs toward a pro-inflammatory, anti-tumoral M1-like phenotype has emerged as a central objective in contemporary cancer immunotherapy [7,8,9].

Among the various strategies, direct genetic engineering of macrophages to generate Chimeric antigen receptor macrophages (CAR-M) provides unmatched antigen specificity and durable therapeutic activity, enabling macrophages to selectively recognize and eradicate cancer cells [10,11,12,13,14,15,16]. Despite its promise, the clinical translation of CAR-M therapy remains fundamentally constrained by a long-standing obstacle: the efficient delivery of genetic cargo into primary macrophages [17,18,19]. Lipid nanoparticles (LNP) have emerged as the leading non-viral vectors for nucleic acid delivery owing to their established clinical success, biocompatibility, and scalability [20,21,22,23]. Their efficiency, nevertheless, declines sharply in quiescent, non-proliferating macrophages, particularly in the M2-polarized subsets. This resistance reflects the interplay of multiple intrinsic barriers, including rigid membrane structures, highly active endo-lysosomal degradation, and innate immune sensing pathways that rapidly eliminate exogenous nucleic acids [24].

Efforts to overcome these limitations have largely centered on tuning the physicochemical properties of LNP-by modifying lipid composition, adjusting surface charge, or adding stealth coatings to prolong circulation and promote uptake [25,26,27,28,29]. While these strategies yield incremental improvements, they frequently reach an efficacy plateau, underscoring that macrophage-intrinsic biology, rather than vehicle design alone, constitutes the dominant barrier. This recognition motivates a paradigm shift: nanocarriers should not only be engineered as robust delivery vehicles but also as modulators capable of transiently altering macrophage physiology to create a more permissive state for transfection. We therefore hypothesized that embedding a bioactive lipid within the LNP structure could endow the system with dual functionality, simultaneously serving as a structural component and as a functional modulator of macrophage state.

To test this concept, we selected arachidonic acid (ARA), a canonical pro-inflammatory ω-6 polyunsaturated fatty acids (PUFA) [30]. As a precursor to bioactive inflammatory mediators, ARA enhances macrophage phagocytosis, promotes pro-inflammatory cytokine production, and modulates membrane dynamics [31,32,33,34,35]—all features associated with a more “activated” phenotype potentially conducive to transfection. Incorporating ARA into the LNP formulation would allow the nanocarrier not only to deliver mRNA payloads but also to functionally prime M2-polarized macrophages upon contact, thereby creating a transiently permissive state that facilitates endosomal escape and gene expression.

**Scheme 1:**
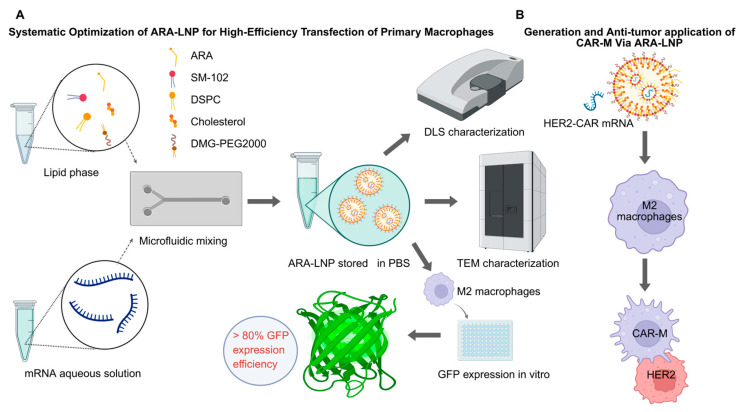
(**A**) Schematic of the two-stage optimization for the bioactive ARA-LNP platform. The strategy involved sequential optimization of (1) the lipid phase, by tuning the molar ratio of the bioactive component ARA, and (2) the aqueous phase, by identifying the optimal mRNA payload concentration. This systematic approach was designed to maximize mRNA delivery and gene expression in primary M2-polarized macrophages for downstream therapeutic applications like CAR-M engineering. (**B**) Schematic of engineered CAR-M generated via ARA-LNP delivery exhibits potent anti-tumor activity in vitro.

Here, we present the rational design, systematic optimization, and validation of an ARA-integrated LNP (ARA-LNP) platform for efficient mRNA delivery into primary M2 macrophages (Scheme 1). We demonstrate that a precisely tuned ARA-LNP dramatically enhances mRNA transfection efficiency, achieving over 80% delivery in otherwise recalcitrant M2-polarized bone marrow-derived macrophages (M2-BMDMs). Building on this capability, we successfully generated human epidermal growth factor receptor 2 (HER2)-targeting CAR-M (HER2-CAR-M), which exhibited potent and specific anti-tumor activity in vitro. Collectively, these findings establish a “bioactive nano vehicle” paradigm, offering a powerful and broadly applicable strategy to overcome the critical delivery bottleneck in macrophage-based cell therapies and to expand opportunities for the genetic engineering of immune cells.

## 2. Results

### 2.1. Influence of Lipid Composition on ARA-LNP Physicochemical Properties and Transfection Efficacy

**Figure 1:**
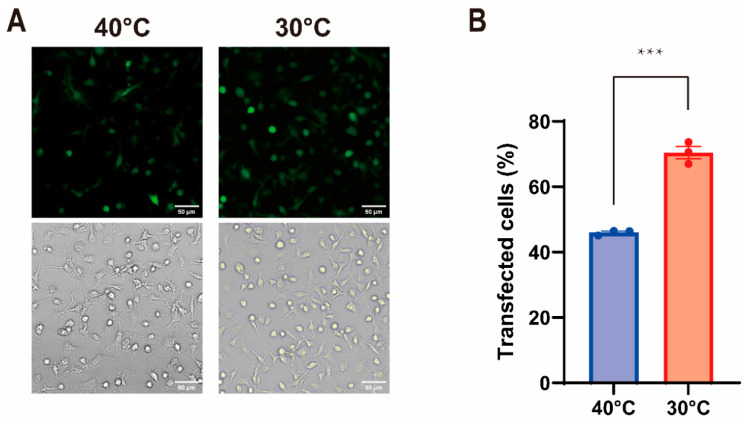
LNP formulation at 30 °C improves transfection efficiency. (**A**) Representative fluorescence micrographs (Scale bars: 50 µm) and (**B**) quantification of transfection efficiency in M2- BMDMs for ARA-LNP prepared at either 30 °C or 40 °C, indicating that the lower temperature is optimal. Data are presented as mean ± SEM. Statistical significance was determined by one-way ANOVA with Tukey’s post-hoc test, *** *p* < 0.001.

To develop an LNP formulation with enhanced transfection efficiency in macrophages, we first investigated the effect of incorporating different ω-6 PUFA, such as ARA, γ-linolenic acid, and linoleic acid, into the lipid phase. The specific lipid formulations are depicted in Supplementary Table S1. All LNP were prepared using standardized microfluidic procedures. When tested in bone marrow-derived M2-type macrophages, ARA-LNP mediated significantly higher reporter protein expression compared to those containing γ-linolenic acid and linoleic acid (Supplementary Figures S1 and S2). Based on this superior performance, ARA was selected as the key bioactive component for further development. Prior to optimizing the ARA molar ratio in the lipid phase, we also evaluated the reaction temperature during microfluidic synthesis. We found that ARA-LNP formulated at 30 °C exhibited superior transfection efficiency in M2-type macrophages compared to those synthesized at the conventional temperature of 40 °C (Figure 1). Thus, 30 °C was established as the standardized formulation temperature for all subsequent experiments.

**Figure 2:**
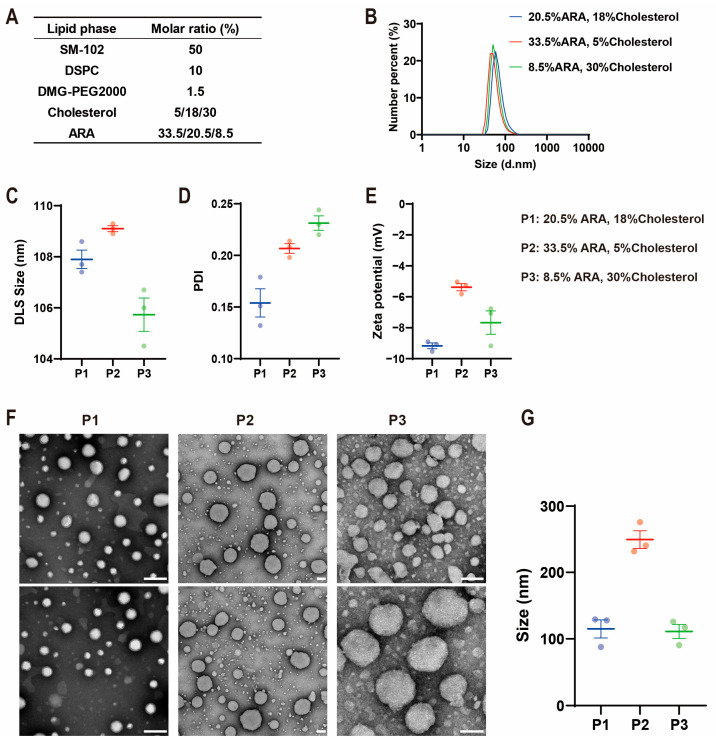
Physicochemical characterization of LNP with varying ARA content. (**A**) Table detailing the molar compositions of the three lipid formulations tested. (**B**) Hydrodynamic diameter and (**C**) corresponding size distribution curves for LNP formulated with 8.5, 20.5, or 33.5 mol% ARA, as measured by DLS. (**D**) PDI values derived from DLS measurements. (**E**) Zeta potential of the three LNP formulations. (**F**) Representative TEM images of the nanoparticles. Scale bars: 200 nm. (**G**) Quantification of nanoparticle diameters from TEM images. Data are presented as mean ± SEM.

Next, we systematically investigated the impact of the ARA molar ratio on the physicochemical properties of the LNP. While the molar percentages of the ionizable lipid (SM-102), helper lipid (DSPC), and PEG lipid (DMG-PEG2000) remained constant, we formulated three LNP variants with increasing ARA content (8.5, 20.5, and 33.5 mol%), compensating with cholesterol to maintain a combined molar percentage of 38% for ARA and cholesterol (Figure 2A). These formulations were used to encapsulate Green Fluorescent Protein (GFP) mRNA. Characterization using dynamic light scattering (DLS) revealed that all formulations possessed comparable hydrodynamic diameters (~105–110 nm) (Figure 2B,C), with the 20.5 mol% ARA formulation displaying the most uniform particle size distribution (Figure 2D). Zeta potential for all formulations was consistent, ranging from −5 to −10 mV, indicating that ARA incorporation had minimal effect on the nanoparticle surface charge (Figure 2E). In addition, for a more precise morphological characterization, transmission electron microscopy (TEM) was employed (Figure 2F). TEM analysis confirmed that LNP with low (8.5 mol%) and medium (20.5 mol%) ARA contents were spherical nanoparticles with diameters around 110 nm. In contrast, the highest ARA content (33.5 mol%) resulted in significantly larger and less uniform particles approaching 250 nm in diameter (Figure 2G). Collectively, these data demonstrate that the molar ratio of ARA is a critical parameter influencing the physicochemical properties of the resulting ARA-LNP.

It is worth noting that TEM imaging revealed the presence of smaller spheroidal structures alongside the predominant ARA-LNP population, indicating a degree of size heterogeneity that is not fully captured by intensity-weighted DLS measurements. Such heterogeneity could influence the amount of mRNA cargo encapsulated per particle and thus impact the uniformity of the therapeutic dose. While this size diversity might facilitate uptake by phagocytic cells, achieving a more monodisperse particle population will be an important consideration for future translational applications.

**Figure 3:**
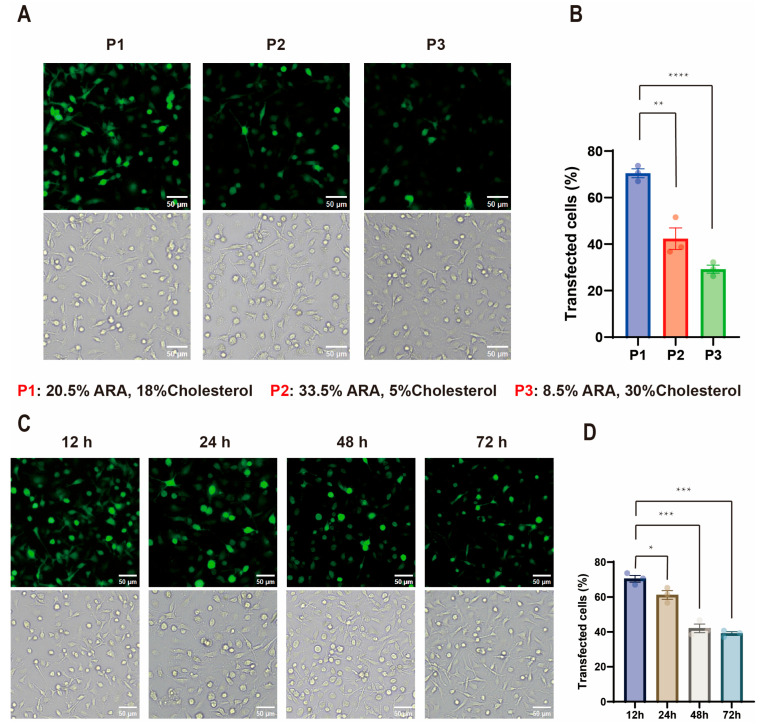
An optimal ARA molar ratio maximizes mRNA transfection efficiency and duration in M2 macrophages. (**A**) Representative fluorescence micrographs of M2-BMDMs 12 h after transfection with LNP containing varying ARA content. Scale bars: 50 µm. (**B**) Quantification of GFP-positive cells, demonstrating that the 20.5 mol% ARA formulation achieved the highest transfection efficiency. (**C**) Representative micrographs showing the persistence of GFP expression over 72 h for the optimal 20.5 mol% ARA-LNP. Scale bars: 50 µm. (**D**) Time-course analysis of transfection efficiency, indicating robust and durable gene expression. Data are presented as mean ± SEM. Statistical significance was determined by one-way ANOVA with Tukey’s post-hoc test. * *p* < 0.05, ** *p* < 0.01, *** *p* < 0.001, **** *p* < 0.0001.

We then assessed the transfection performance of these formulations in M2-BMDMs (Figure 3A). Notably, the 20.5 mol% ARA-LNP formulation yielded the highest GFP expression efficiency (70.5%), outperforming the other formulations (Figure 3B). This result revealed a non-monotonic relationship, where transfection efficiency improved with increasing ARA content up to an optimal threshold (20.5 mol%), beyond which it declined. To evaluate the kinetics and durability of gene expression mediated by this lead formulation, M2-BMDMs were transfected and monitored over time. Expression peaked at 12 h post-transfection (70.5% GFP-positive cells) and, while gradually declining, remained robust, with over 60% of cells expressing GFP at 24 h and a substantial 39.1% remaining positive at 72 h (Figure 3C,D). These results confirmed that incorporating 20.5 mol% ARA significantly enhanced both the efficiency and persistence of gene expression in these hard-to-transfect primary macrophages. These findings underscore that lipid composition is a critical determinant of transfection efficacy. Based on its superior performance, the 20.5 mol% ARA formulation was selected as the lead candidate for further optimization.

### 2.2. Influence of mRNA Concentration on ARA-LNP Properties and Transfection Efficacy

**Figure 4:**
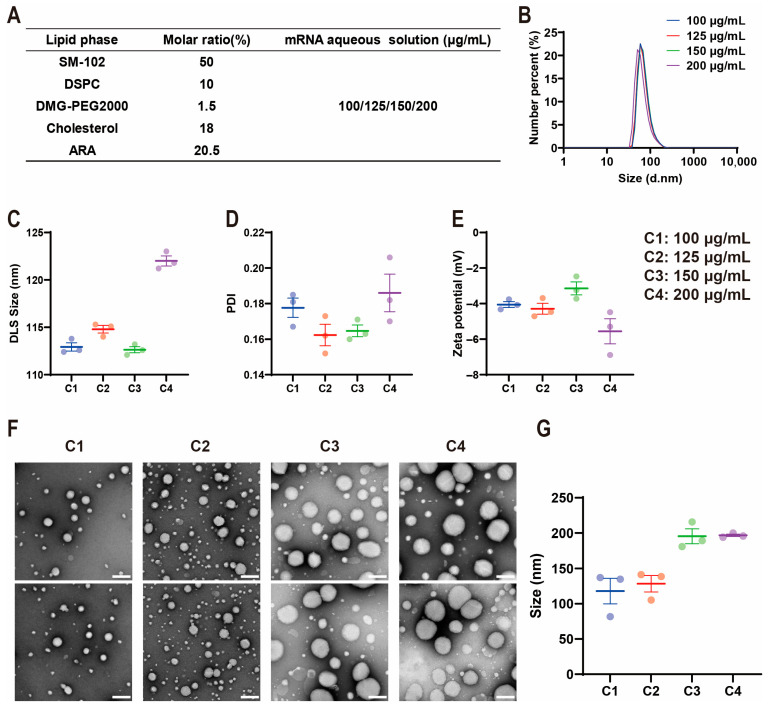
Influence of mRNA concentration on the physicochemical properties of ARA-LNP. (**A**) Table detailing the constant lipid phase composition and varied aqueous phase mRNA concentrations used for LNP formulation. (**B**) Hydrodynamic diameter and (**C**) size distribution curves of ARA-LNP prepared with mRNA concentrations ranging from 100 to 200 µg/mL. (**D**) PDI values for each formulation. (**E**) Zeta potential measurements. (**F**) Representative TEM images. Scale bars: 200 nm. (**G**) Quantification of nanoparticle diameters from TEM, showing a non-linear increase in size with mRNA concentration, plateauing at 150 µg/mL. Data are presented as mean ± SEM.

Having established the optimal lipid composition, we next sought to optimize the mRNA payload by investigating the effect of mRNA concentration in the aqueous phase. Using the lead 20.5 mol% ARA lipid mixture, we prepared four LNP batches by varying the initial GFP mRNA concentration (100, 125, 150, and 200 µg/mL) in the sodium acetate buffer (Figure 4A). DLS measurements revealed that hydrodynamic diameter remained relatively stable at lower mRNA concentrations, with a slight increase observed only at the highest concentration of 200 µg/mL (Figure 4B,C). The polydispersity index (PDI) for all formulations remained below 0.2 (Figure 4D), indicating the formation of monodisperse nanoparticles across the tested concentration range. Similarly, the zeta potential showed only a minor shift towards a more negative charge with increasing mRNA content, from −4.05 ± 0.16 mV to −5.56 ± 0.71 mV (Figure 4E). In contrast to DLS, TEM imaging revealed a more pronounced, non-linear increase in particle size with escalating mRNA concentration (Figure 4F,G). The particle size increased from 118.02 nm at 100 µg/mL to a maximum of 195.46 nm at 150 µg/mL, then plateaued, suggesting the saturation of the LNP’s encapsulation capacity at a critical mRNA-to-lipid ratio [36].

**Figure 5:**
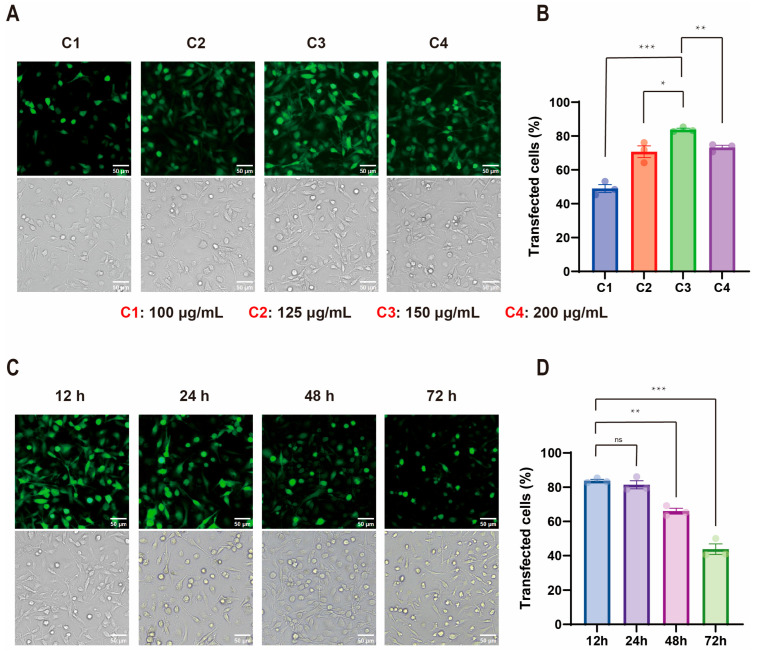
Optimal mRNA payload concentration leads to peak transfection efficiency in M2 macrophages. (**A**) Representative fluorescence micrographs of M2-BMDMs 12 h after transfection with ARA-LNP formulated with different mRNA concentrations. Scale bars: 50 µm. (**B**) Quantification of transfection efficiency, identifying 150 µg/mL as the optimal mRNA concentration, achieving >80% GFP-positive cells. (**C**) Representative micrographs illustrating the time course of GFP expression for the fully optimized ARA-LNP formulation (150 µg/mL mRNA). Scale bars: 50 µm. (**D**) Quantification of transfection efficiency over 72 h, demonstrating sustained high-level gene expression. Data are presented as mean ± SEM. Statistical significance was determined by one-way ANOVA with Tukey’s post-hoc test, ^ns^ *p* > 0.05, * *p* < 0.05, ** *p* < 0.01, *** *p* < 0.001.

**Figure 6:**
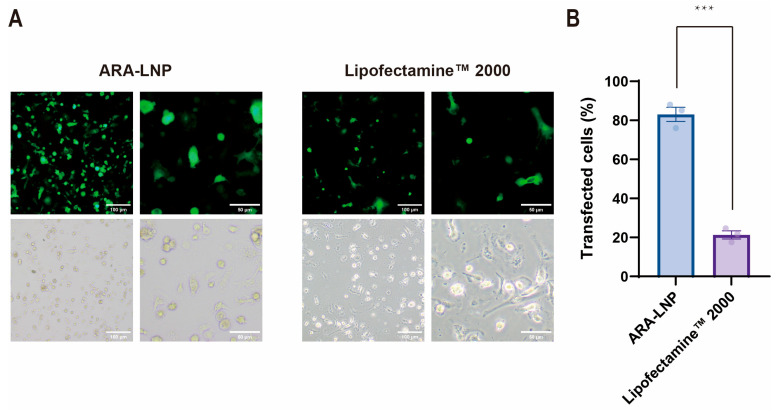
Comparison of transfection efficiency between optimized ARA-LNP/GFP mRNA and commercial reagent Lipofectamine™ 2000/GFP mRNA in M2 macrophages. (**A**) Representative fluorescence micrographs of M2-BMDMs transfected with GFP mRNA using either the optimized ARA-LNP or Lipofectamine™ 2000. Scale bars: 100 µm (left), 50 µm (right). (**B**) Quantification of GFP-positive cells, demonstrating the approximately four-fold higher efficiency of the ARA-LNP platform in these hard-to-transfect cells. Data are presented as mean ± SEM. Statistical significance was determined by one-way ANOVA with Tukey’s post-hoc test, *** *p* < 0.001.

Subsequently, we evaluated the impact of mRNA concentration on transfection efficiency in M2-BMDMs. A clear dose-dependent increase in GFP expression was observed, culminating in a striking peak efficiency of 83.76% with LNP formulated using 150 µg/mL mRNA (150 µg/mL GFP mRNA-ARA-LNP) (Figure 5A,B). However, further increasing the concentration to 200 µg/mL resulted in a notable decrease in transfection, defining 150 µg/mL as the optimal concentration. We then compared the transfection efficiency between the optimized 150 µg/mL GFP mRNA-ARA-LNP and the commercial liposomal agent Lipofectamine™ 2000/GFP mRNA in M2 macrophages, revealing an approximately four-fold higher efficiency with the former. This demonstrated that our study successfully developed an LNP delivery system with high transfection efficacy for M2-type macrophages, effectively surmounting the transfection efficiency bottleneck of existing gene delivery vectors in primary macrophages (Figure 6). Furthermore, to evaluate the persistence of gene expression mediated by the optimized formulation in M2-type macrophages, we treated cells with 150 µg/mL GFP mRNA-ARA-LNP and assessed the transfection efficiency at various timepoints (Figure 5C,D). It was noted that, although transfection efficiency exhibited a gradual decline over time, the proportion of GFP-positive cells at 24 h post-transfection was only 2.3% lower than the peak level observed at 12 h. Remarkably, even at the 72 h timepoint, a substantial 45.3% of cells remained GFP-positive. These findings indicated that the ARA-LNP enables durable gene expression, thereby laying the groundwork for enhanced efficacy in CAR-M therapy. Taken together, we found that an optimal mRNA concentration in the aqueous phase is a crucial determinant of transfection efficiency in M2-type macrophages. This observed optimum may have significant implications regarding the size of the resultant LNP, with ARA-LNP formulated at 150 µg/mL mRNA demonstrating the most robust GFP expression.

In summary, through a two-stage optimization process, we identified a lead ARA-LNP formulation with exceptional performance. We began our investigation by systematically evaluating lipid phase compositions, a critical reaction component in LNP formulation. By adjusting the molar percentage of ARA in the lipid phase, we prepared three representative ARA-LNP with varying ARA ratios. After assessing their physicochemical properties along with transfection efficiency in bone marrow-derived M2-type macrophages, 20.5 mol% ARA was determined as the optimal lipid phase composition for further optimization. Additionally, we also considered an additional key factor influencing transfection efficiency-mRNA concentration. By preparing four ARA-LNP formulations with different mRNA concentrations in sodium acetate buffer solution, we investigated their characteristics and transfection performance in bone marrow-derived M2-type macrophages. This analysis identified 150 μg/mL as the optimal mRNA concentration for the aqueous phase.

The final optimized ARA-LNP formulation for M2-type macrophage transfection was as follows: the lipid phase consisted of an ethanolic solution containing 50 mol% SM-102, 10 mol% DSPC, 1.5 mol% DMG-PEG2000, 18 mol% cholesterol and 20.5 mol% ARA; the aqueous phase comprised a 150 μg/mL mRNA solution in sodium acetate buffer (pH 5.0). The resulting ARA-LNP loaded with GFP mRNA exhibited a zeta potential of −5.27 ± 0.36 mV, spherical nanoparticles of 195.46 nm, and achieved GFP expression levels of up to 83.76% in primary bone marrow-derived M2-type macrophages. This ARA-LNP laid a robust foundation for developing CAR-M therapeutics in vitro and in vivo, thereby enhancing antitumor immunotherapy.

### 2.3. In Vitro Generation and Anti-Tumor Efficacy Validation of CAR-M Macrophages via ARA-LNP Delivery

**Figure 7:**
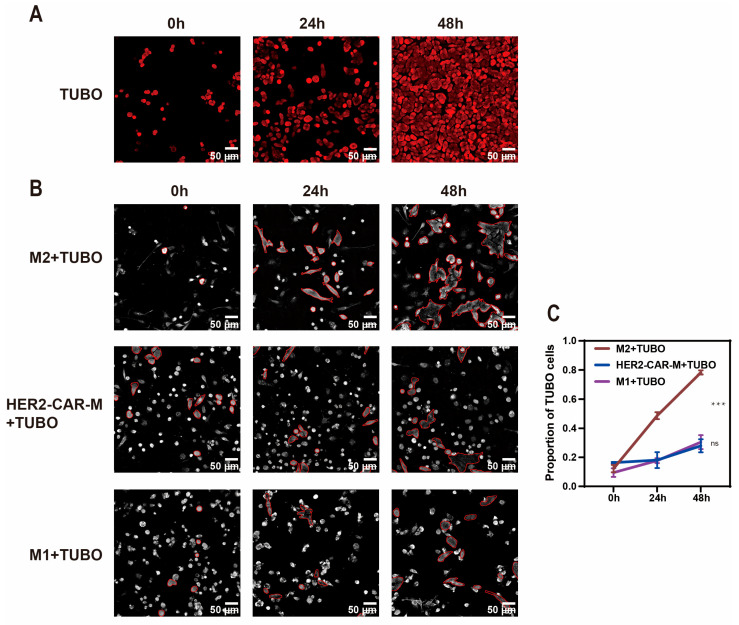
Engineered CAR-M generated via ARA-LNP delivery exhibits potent anti-tumor activity in vitro. (**A**) Proliferation of HER2-expressing TUBO tumor cells cultured alone over 48 h. (**B**) Representative time-lapse holotomographic images of TUBO cells co-cultured with M2 macrophages (negative control), HER2-CAR-M, or M1 macrophages (positive control) at an E:T ratio of 10:1. The cells circled in red are TUBO cells, and the others are macrophages. Scale bars: 50 µm. (**C**) Quantification of the TUBO cell proportion relative to the total cell numbers over time. HER2-CAR-M cells effectively suppress tumor growth to a degree comparable to M1 macrophages, whereas M2 macrophages do not. Data are presented as mean ± SEM. Statistical significance was determined by one-way ANOVA with Tukey’s post-hoc test, ^ns^ *p* > 0.05, *** *p* < 0.001.

**Figure 8:**
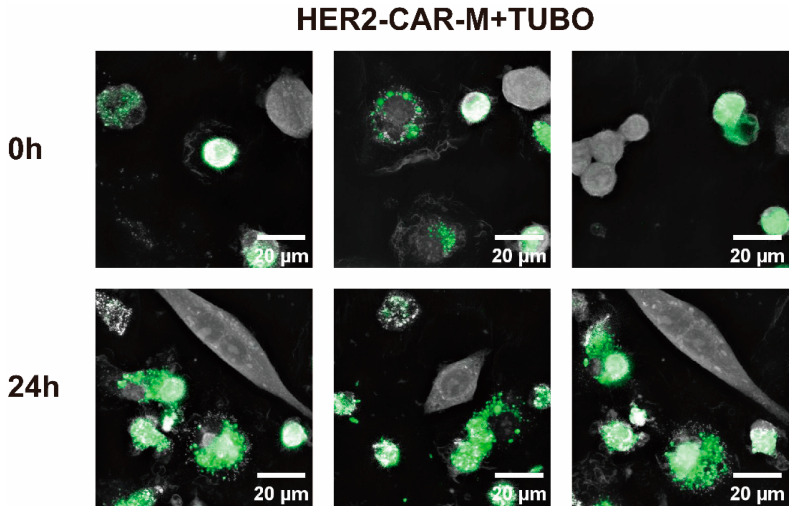
Morphological distinction between HER2-CAR-M and TUBO tumor cells in co-culture. Holotomographic fluorescence micrographs at 0 h and 24 h. HER2-CAR-M are identifiable by their expression of a GFP-tagged CAR construct (green) and an irregular morphology with cellular protrusions. In contrast, TUBO cells are non-fluorescent and exhibit a more uniform, rounded, or elongated shape, allowing for unambiguous identification and quantification of each cell type. Scale bars: 20 µm.

Having developed a highly efficient LNP platform, we next sought to determine its utility for a therapeutically relevant application: the generation of functional CAR-M. We encapsulated mRNA encoding a second-generation CAR targeting the HER2 into our optimized ARA-LNP. These were used to transfect M2-BMDMs, generating HER2-CAR-M cells. The anti-tumor functionality of these engineered macrophages was evaluated in a co-culture assay with HER2-expressing TUBO mouse mammary carcinoma cells at an effector-to-target (E:T) ratio of 10:1. We monitored tumor cell fate over 48 h using a nanolive 3D holotomographic microscope, comparing the effects of HER2-CAR-M cells against unmodified M2 macrophages (negative control) and pro-inflammatory M1 macrophages (positive control for cytotoxicity). As expected, TUBO cells proliferated rapidly when cultured alone, reaching near-confluency within 48 h (Figure 7A). When co-cultured with control M2 macrophages, they also expanded without inhibition, with the distinct cell morphologies readily distinguishable (Figure 7B and Figure 8). After 48 h, TUBO cells constituted 78.33% of the total cell population in the M2 co-culture, confirming that unmodified M2 macrophages failed to exert an anti-tumor effect (Figure 7C). In stark contrast, the HER2-CAR-M cells effectively suppressed tumor proliferation. In these co-cultures, the proportion of TUBO cells, which started at approximately 16%, remained remarkably stable, reaching only 27% after 48 h. This potent anti-tumor activity was comparable to that of the M1 macrophage positive control group. These results provide compelling evidence that our ARA-LNP platform can successfully deliver functional genetic payloads to primary macrophages, enabling the engineering of potent CAR-M effectors capable of specific tumor cell recognition and elimination.

## 3. Discussion

In this study, we addressed the critical challenge of inefficient gene delivery to primary macrophages, a major roadblock in the development of macrophage-based immunotherapies such as CAR-M. To overcome this barrier, we introduced and validated a “bioactive nanocarrier” strategy by engineering lipid nanoparticles that incorporate ARA as a functional structural component. Through systematic optimization of both the lipid composition and mRNA payload, we developed an ARA-LNP formulation that achieved an exceptional transfection efficiency of over 80% in M2-BMDMs, a notoriously difficult-to-transfect cell type that models the immunosuppressive TAMs found in tumors. Importantly, we demonstrated the therapeutic potential of this platform by generating HER2-CAR-M that exhibited potent and antigen-specific anti-tumor activity in vitro. These findings not only provide a robust new tool for macrophage engineering but also establish a conceptual advance in nanocarrier design, where the delivery vehicle itself actively modulates the target cell to overcome intrinsic biological barriers.

Our approach diverges from conventional LNP optimization, which has historically focused on passive physicochemical parameters such as particle size, surface charge, and stealth coatings. While these properties remain critical, our findings highlight the substantial gains achievable by actively engaging with the target cell’s biology. The selection of ARA was hypothesis-driven, based on its known pro-inflammatory and membrane-modulating activities [37,38]. We speculate that the remarkable improvement in transfection efficiency stems from a multi-pronged mechanism. First, the incorporation of the kinked, polyunsaturated ARA molecule likely enhances the fluidity of the LNP lipid bilayer, thereby facilitating more efficient fusion with the endosomal membrane and promoting endosomal escape-a key bottleneck in LNP-mediated delivery. Second, upon uptake, ARA released within the macrophage could transiently trigger pro-inflammatory signaling pathways, creating a cellular state more conducive to the processing and translation of foreign mRNA, potentially by altering metabolic fluxes or downregulating innate immune sensing pathways that would otherwise degrade the payload. The non-linear relationship between ARA content and transfection efficiency, where performance peaked at 20.5 mol% and declined thereafter, suggests a delicate balance. Excessive ARA may lead to nanoparticle instability, as hinted by the larger, less uniform particles observed at 33.5 mol%, or induce cellular toxicity, thereby compromising transgene expression.

The successful generation of functional CAR-M underscores the translational significance of our platform. By achieving high-efficiency transfection, ARA-LNP make it possible to generate large numbers of therapeutically active macrophages from primary cells without the use of viral vectors or extensive pre-conditioning protocols. Notably, the engineered CAR-M demonstrated robust tumoricidal activity comparable to that of pro-inflammatory M1 macrophages. This suggests that the CAR-mediated activation signal is sufficient to override the baseline M2 polarization, effectively reprogramming these cells into tumoricidal effectors upon antigen encounter.

While this study provides a strong proof-of-concept, several avenues warrant future investigation. A deeper mechanistic exploration is needed to precisely delineate how ARA enhances transfection. Studies employing lipidomics, transcriptomics, and metabolic profiling (e.g., Seahorse assays) would help confirm our hypothesis regarding membrane fusion and metabolic reprogramming. Furthermore, while we demonstrated robust in vitro efficacy, the true potential of this technology must be validated in vivo. Future work will focus on assessing the biodistribution, safety, and therapeutic efficacy of systemically or locally administered ARA-LNP in preclinical solid tumor models. Another intriguing direction is to examine whether the pro-inflammatory properties of ARA synergistically remodel the tumor microenvironment by recruiting other immune cells. Finally, the “bioactive nanocarrier” concept introduced here with ARA could be extended to a broader class of immunomodulatory lipids or small molecules. Incorporating molecules that, for example, inhibit lysosomal acidification or modulate specific innate immune sensors could provide alternative or complementary strategies to enhance gene delivery in primary macrophages and other challenging immune cell types like dendritic cells or neutrophils.

In conclusion, this work presents a paradigm-shifting approach to nanocarrier design that bridges materials science and immunology. By embedding intrinsic biological activity into the delivery vehicle, we created a potent platform that effectively overcomes the inherent resistance of primary macrophages to genetic engineering. This technology holds significant promise for advancing CAR-M therapy and offers a versatile blueprint for the development of next-generation delivery systems for a wide range of immunotherapeutic applications.

## 4. Materials and Methods

### 4.1. Materials

Arachidonic acid, γ-linolenic acid andlinoleic acid was obtained from Shanghai Aladdin Biochemical Technology Co., Ltd., Shanghai, China. 1-Octylnonyl 8-[(2-hydroxyethyl) [6-oxo-6-(undecyloxy) hexyl] amino]-octanoate (SM-102), cholesterol, 1,2-dimyristoyl-rac-glycerol-3-methoxypolyethylene glycol-2000 (DMG-PEG2000) were purchased from Xiamen Sinopeg Biotechnology Co., Ltd., Xiamen, China. 1,2-dioctadecanoyl-sn-glycero-3-phophocholine (DSPC) was purchased from Avanti Polar Lipids, Alabaster, AL, USA. GFP mRNA was from Yeasen Biotechnology Co., Ltd, Shanghai, China. The HER2-targeting CAR genetic constructs (HER2-CAR) plasmids were synthesized by Sangon Biotech Co., Ltd, Shanghai, China, followed by in vitro synthesis of HER2-CAR mRNA in our laboratory. Dulbecco’s Modified Eagle Medium (DMEM), RPMI 1640 Medium and Fetal Bovine Serum (FBS) were purchased from Gibco (Waltham, MA, USA). Murine M-CSF and Recombinant Murine lL-4 were purchased from Pepro Tech, Cranbury, NJ, USA).

### 4.2. The Preparation of LNP

LNP synthesis was performed using microfluidic technology (Micro&Nano Instrument Technology Co., Ltd., Shanghai, China, INanoE). The lipid phase, dissolved in ethanol, comprised the following components at specified molar ratios: SM-102 (50 mol%), DSPC (10 mol%), DMG-PEG2000 (1.5 mol%), cholesterol and PUFA (accounting for 38.5 mol% in total). The aqueous phase consisted of mRNA in sodium acetate buffer (pH 5.0). LNP were formulated by mixing the lipid and aqueous phases at a volumetric ratio of 1:3, with a total flow rate of 16 mL/min. The reaction temperature was adjusted based on experimental requirements. Post-synthesis, the LNP were purified and concentrated using a 30 kDa molecular weight cutoff (MWCO) centrifugal filter device with PBS. The purified LNP were stored at 4 °C for subsequent studies.

### 4.3. The Characterization of LNP

Lipid nanoparticle (LNP) characterization was performed by measuring hydrodynamic diameter, polydispersity index (PDI), and zeta potential using dynamic light scattering (DLS), while morphological analysis was measured with via transmission electron microscopy (TEM). For TEM sample preparation, a 4 μL aliquot of each specimen was deposited onto a carbon-coated copper grid, followed by particle settlement for 45 s, and then stained with 4 μL of phosphotungstic acid solution, incubated for 45 s The samples were air-dried for 3–5 min before microscopic observation.

### 4.4. Isolation, Polarization, and Expansion Culture of Primary Bone Marrow-Derived Monocytes

Primary bone marrow-derived monocytes were isolated from femurs and tibiae of 8–10-week-old female mice. The cells were polarized into M1-like or M2-like macrophage precursors by routine 7-day culture in RPMI-1640 complete medium containing either 40 ng/mL GM-CSF or 20 ng/mL M-CSF, respectively. Terminal differentiation was then achieved through 48-h stimulation at 37 °C under 5% CO_2_ humidified conditions with specific cytokine cocktails: M1-type macrophages were induced by 100 ng/mL Lipopolysaccharides (LPS), 50 ng/mL IFN-γ and 40 ng/mL GM-CSF, while M2-type macrophages were induced by 20 ng/mL IL-4 and 20 ng/mL M-CSF.

### 4.5. Validation of LNP Transfection Efficiency

Bone marrow-derived M2-type macrophages were seeded in 96-well plates at a density of 1 × 10^5^ cells per well and cultured in RPMI-1640 medium. After 24-h incubation, LNP/GFP mRNA complexes containing identical GFP mRNA quantities (1 μg) were added to each well for co-culture. After 12-h incubation, Cells were washed twice thoroughly with PBS to remove residual LNP/GFP mRNA complexes and subsequently maintained in fresh RPMI-1640 medium until the 72 h timepoint. Fluorescence and bright-field images of each delivery system were acquired by fluorescence microscopy following 12-h incubation. Transfection efficiency was quantified as the ratio of GFP-positive cells (fluorescence images) to total cells (bright-field images) using ImageJ (version 1.54g) with threshold-based counting and manual verification and five random regions (0.718 mm × 0.477 mm) per image were analyzed to ensure robust sampling.

### 4.6. Development of an ARA-LNP-Based In Vitro CAR-M Platform and Validation of Its Anti-Tumor Efficacy

For the blank control group, 2.5 × 10^4^ TUBO cells were seeded in nanolive 3D Cell Explorer-compatible imaging dishes. Holotomographic images were acquired using the nanolive 3D real-time imager at 0 h, 24 h, and 48 h post-seeding. Bone marrow-derived M2-type macrophages were transfected with optimized ARA-LNP encapsulating the HER2-CAR mRNA for 24 h, resulting in HER2-CAR-M cells. Subsequently, 2.5 × 10^5^ HER2-CAR-M cells were seeded in nanolive-compatible dishes and cultured for 12 h, followed by co-incubation with 2.5 × 10^4^ TUBO cells. Images were acquired at 0 h, 24 h, and 48 h after initiating co-culture using the nanolive imager. Identical procedures, including cell quantities, pre-culture duration, imaging timepoints, and imaging specifications were performed for the other two effector cell types: bone marrow-derived M2-type macrophages and bone marrow-derived M1-type macrophages co-cultured with TUBO cells. Populations (macrophages vs. TUBO cells) were distinguished by distinct morphological features in high-magnification holotomographic images, validated by inherent GFP fluorescence of HER2-CAR-M (absent in TUBO). Manual cell counting using ImageJ was performed across multiple fields per condition. The proportion of TUBO cells relative to the total cell count was calculated. This combined approach (morphological assessment, fluorescence verification, manual counting) ensures accurate quantification in co-culture assays.

### 4.7. Statistical Analysis

All statistical analyses were conducted using GraphPad Prism (version 10.4.2) and Image J (version 1.54g) software. Data are presented as mean ± SEM. One-way ANOVA was employed to assess statistical differences between experimental groups. A threshold of *p* < 0.05 was defined as the minimum level for statistical significance. All schematic diagrams were generated with BioRender (https://www.biorender.com/, accessed on: 21 August 2025).

## Data Availability

The data that support the findings of this study are available from the corresponding author upon reasonable request.

## Supplementary Materials

The following supporting information can be downloaded at: https://www.mdpi.com/article/10.3390/ijms26189199/s1.
