# Supplementary material for: A Bioactive Lipid Nanoparticle Integrating Arachidonic Acid Enables High-Efficiency mRNA Delivery and Potent CAR-Macrophage Engineering
Source: Int J Mol Sci. 2025 Sep 20;26(18):9199. doi: 10.3390/ijms26189199 (PMC12470929; doi:10.3390/ijms26189199)
Supplement: Supplementary file 1 [file ijms-26-09199-s001.zip › ijms-3859700-supplementary.pdf]

Supporting Information for

A Bioactive Lipid Nanoparticle Integrating Arachidonic Acid Enables High-Efficiency mRNA Delivery and Potent CAR-Macrophage Engineering

Jia Fu[1]，Yanan Zhang[1,2]，Yifan Lv[1]，Ruilin Li[1]，Hongchen Gu[1,2*] and Jingxing Yang[1,2*]

1 School of Biomedical Engineering, Med-X Research Institute, Shanghai Jiao Tong University, Shanghai 200030, China.

2 Hefei Institute of Innovative Early Cancer Screening Technologies, Shanghai Jiao Tong University, Anhui 230000，China.

*Co-correspondence to:

Hongchen Gu: School of Biomedical Engineering, Med-X Research Institute, Shanghai Jiao Tong University, Shanghai 200030, China. Tel/FAX: 86-021-62933323. e-mail: hcgu@sjtu.edu.cn；

*Correspondence to:

Jingxing Yang: School of Biomedical Engineering, Med-X Research Institute, Shanghai Jiao Tong University, Shanghai 200030, China. Tel/FAX: 86-021-62933323. e-mail: tom123yang@sjtu.edu.cn

Table S1. Lipid compositions of LNPs incorporating ARA, γ-linolenic acid, or linoleic acid.

Lipid phase	Molar ratio (%)
SM-102	50
DSPC	10
DMG-PEG2000	1.5
Cholesterol	18
Omega-6 PUFA	20.5

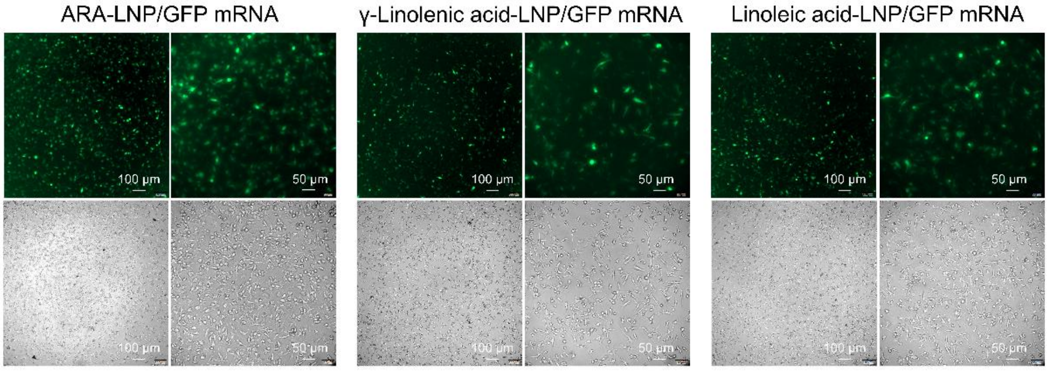

Figure S1. Representative fluorescence micrographs in M2-polarized BMDMs, showing the superior performance of ARA-LNPs. Scale bars: 100 μm (left), 50 μm (right).

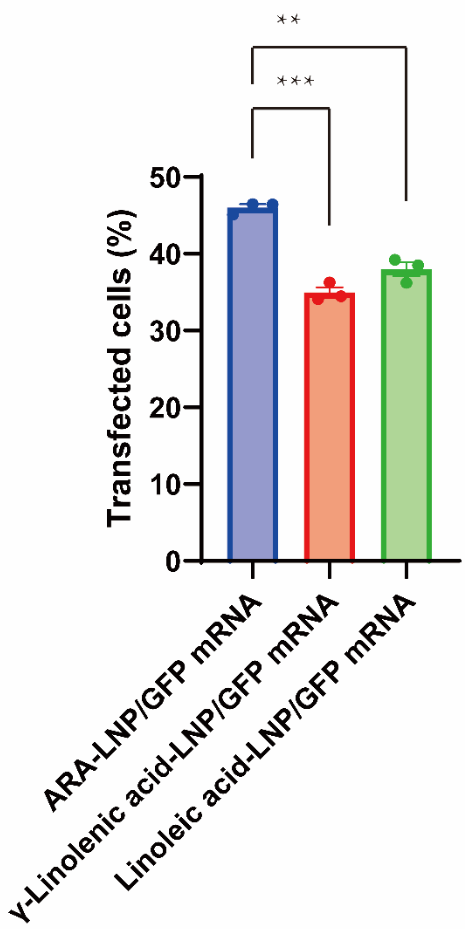

Figure S2. Quantification of transfection efficiency in M2-polarized BMDMs, showing the superior performance of ARA-LNPs. Data are presented as mean ± SEM. Statistical significance was determined by one-way ANOVA with Tukey's post-hoc test, $^{**}p < 0.01$, $^{***}p < 0.001$.
